# MUF-n-Octadecane Phase-Change Microcapsules: Effects of Core pH and Core–Wall Ratio on Morphology and Thermal Properties of Microcapsules

**DOI:** 10.3390/molecules29204794

**Published:** 2024-10-10

**Authors:** Lin Lin, Ziqi Li, Jian Zhang, Tonghua Ma, Renzhong Wei, Qiang Zhang, Junyou Shi

**Affiliations:** 1 School of Agricultural Engineering and Food Science, Shandong University of Technology, Zibo 255000, China; 2 Key Laboratory of Wooden Materials Science and Engineering of Jilin Province, Beihua University, Jilin 132013, China; 3 Treezo Group New Material Technology Co., Ltd., Hangzhou 311112, China

**Keywords:** phase change, microcapsules, emulsifier, latent heat, encapsulate

## Abstract

Phase change energy storage microcapsules were synthesized in situ by using melamine-formaldehyde–urea co-condensation resin (MUF) as wall material, n-octadecane (C18) as core material and styryl-maleic anhydride copolymer (SMA) as emulsifier. Fourier transform infrared spectroscopy, scanning electron microscopy, differential scanning calorimetry and thermogravimetric analysis were used to study the effects of emulsifier type, emulsifier dosage, core–wall ratio and pH on the morphology and thermal properties of microcapsules. The results show that the pH of core material and the ratio of core to wall have a great influence on the performance of microcapsules. SMA emulsifiers and MUF are suitable for the encapsulation of C18. When the pH is 4.5 and the core–wall ratio is 2/1, the latent heat and encapsulation efficiency of phase transition reaches 207.3 J g^−1^ and 84.7%, respectively. The prepared phase-change microcapsules also have good shape stability and thermal stability.

## 1. Introduction

In the process of phase transformation, organic solid–liquid phase variable materials can store or release a large amount of latent heat at almost isothermal temperature. At present, a variety of organic solid–liquid phase variable materials have been used for thermal energy storage, such as organic alkanes, organic fatty acids, polyols and so on. Among these materials, organic alkanes are very popular due to their many advantages, including high energy storage density, good chemical stability, non-corrosion, non-toxicity, non-phase separation and low subcooling, making them ideal materials for many heat storage applications [1,2]. However, leakage in the solid–liquid phase transformation process is an inherent defect of the phase change energy storage microcapsules (PCM), which greatly limits its application range. The use of microencapsulation technology to encapsulate phase-change materials in a sealed tiny container to make core–shell structure phase-change microcapsules can effectively prevent the leakage of phase-change materials and improve their shape stability [3].

Microencapsulation not only enables the PCM to maintain its macroscopic solid state when the solid–liquid phase changes, but also provides sufficient protection for the PCM from harmful interactions and interference of the surrounding environment [4,5,6]. In addition, microencapsulation can provide more heat transfer area for the phase-change material, resulting in significantly enhanced heat transfer and thermal response [7]. Therefore, microencapsulation has been recognized as a reliable phase-change material encapsulation technology. The shell material used to encapsulate the phase-change material should meet the requirements of no chemical reactivity with the phase-change material, good sealing, good chemical stability and thermal stability so as to provide good shape stability for the phase-change material. At present, many studies have been carried out on the encapsulation of phase-variant materials with various shell materials, including melamine-formaldehyde–urea co-condensation resin (MUF), melamine-formaldehyde resin (MF), urea-formaldehyde resin (UF), polystyrene (PS), polymethyl methacrylate (PMMA), silicon dioxide (SiO_2_), titanium dioxide (TiO_2_), calcium carbonate (CaCO_3_), etc. [8,9,10,11,12,13]. Compared with the case materials, poor sealing of inorganic materials may lead to leakage of phase-change materials. Therefore, organic materials are ideal shells for encapsulating phase-change materials [14]. In the case of materials, MUF is widely used to encapsulate phase-change materials because of its good sealing, thermal stability and excellent waterproof performance. MUF also has good mechanical properties and a certain toughness [15,16,17]. In addition, the MUF synthesis process is simple and the price is reasonable, so it has a broader prospect in large-scale applications. Wang et al. [18] prepared microcapsule phase-change materials by coating paraffin with nano Fe_3_O_4_-modified melamine urea formaldehyde resin (MUF). Similarly, Han et al. [19] prepared phase-change microcapsules with paraffin as the core material and MUF as the shell.

In the preparation of phase-change microcapsules, the control of the pH value is very important because it affects the emulsification process, polymerization reaction and the properties of the final product. During the emulsification process, the pH value will affect the performance of the emulsifier and the stability of the emulsion. For different emulsifiers, there is a specific optimal pH range. For example, some ionic emulsifiers have better surface activity in a specific pH range so that a more stable emulsion can be formed. In acidic environments, MF prepolymers are positively charged due to the reaction of hydroxyl methyl groups with hydrogen ions. The positively charged prepolymer is strongly attracted to the nuclear particles with a negative electron field by an anionic SMA emulsifier, and under the action of acid and heat, a microencapsulated reaction occurs on the surface of the nuclear particles to form a microencapsulated film. In the system containing the cationic SMA emulsifier, MF prepolymer is repelled by core particles with the same positive charge and does not enclose the core material. In the non-ionic SMA emulsifier system, the positively charged MF prepolymer is partially attracted to the core particle surface, and the encapsulation is not complete. In the system without the emulsifier, due to the weak hydrogen bond, a small amount of MF prepolymer interacts with the hydroxyl group and is deposited on the surface of core particles [20]. In the interfacial polymerization process, the pH value affects the ionization state of the monomer, thus affecting the rate and efficiency of the polymerization reaction [21]. For example, when using polyamide or polyurethane as a wall material, the pH value needs to be controlled in the acidic or alkaline range to facilitate the reaction. In in situ polymerization, pH values affect the solubility and reaction rate of monomers [22]. Chang et al. [23] synthesized phase-change microcapsules using N-tetradecane as the core material and SDS as the emulsifier and concluded that the microcapsules prepared when the mass fraction of SDS was 1.2 wt%, the pH was 3.5 and the core–shell mass ratio was 2:1 had the best performance. Huang et al. [24] prepared carboxymethyl cellulose-modified phase-change microcapsules with urea–formaldehyde resin as the shell, and explored the effects of curing the pH value, emulsifier dosage and emulsification rate on the properties of microcapsules.

The core–wall ratio refers to the mass ratio of the phase-change material to the wall material in microcapsules. Generally speaking, the higher the core material content, the greater the latent heat of the phase transition microcapsules, and the higher core–wall ratio means that the microcapsules contain more PCM, thus increasing the energy storage capacity. However, too high a core–wall ratio may lead to too thin a wall material, which makes it easy for the PCM to break or leak during use, affecting the structural stability and mechanical strength of microcapsules [25,26]. The thermal conductivity of the phase-change material is usually higher than that of the wall material, and a higher core–wall ratio usually improves the thermal conductivity of the microcapsule, but if the wall material is too thin, it may not be able to effectively isolate the temperature change of the external environment, resulting in the phase-change material releasing or absorbing heat too quickly and failing to achieve the desired temperature control effect [27].

**Figure 1:**
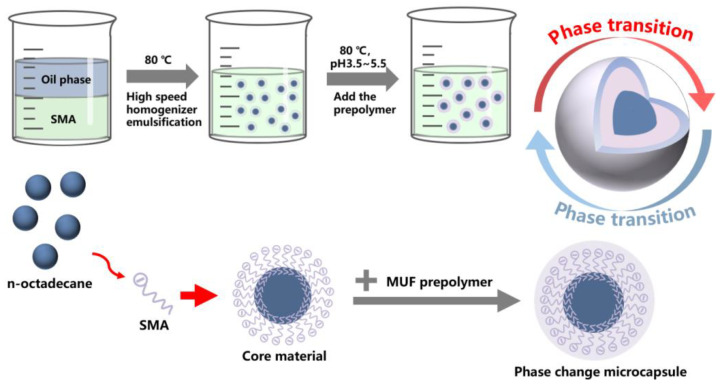
Schematic diagram of MUF-n-octadecane phase-change microcapsules.

Many scholars have improved the performance of the phase-change microcapsules by modifying the shell material, adding thermal conductivity enhancers and changing the packaging method, but they may have overlooked the most basic synthesis process of microcapsules. Based on the synthesis process of microcapsules, it is possible to better improve their defects and seek the changing patterns of microcapsule morphology, latent heat, shape stability, etc. In this work, the effects of emulsifier type, emulsifier dosage, core–shell ratio and pH on the morphology and thermal properties of microcapsules were studied. As shown in Figure 1, phase-change microcapsules of C18 with a MUF shell were successfully prepared. The morphology of the C18 phase-change microcapsules prepared by different technologies was mainly observed by scanning electron microscopy, and their latent heat was carefully analyzed by DSC. The phase-change microcapsules prepared in this work are compatible with building materials and can be applied in building thermal storage fields such as exterior wall insulation and building roofs.

## 2. Results

### 2.1. Microcapsule Morphology Analysis

**Figure 2:**
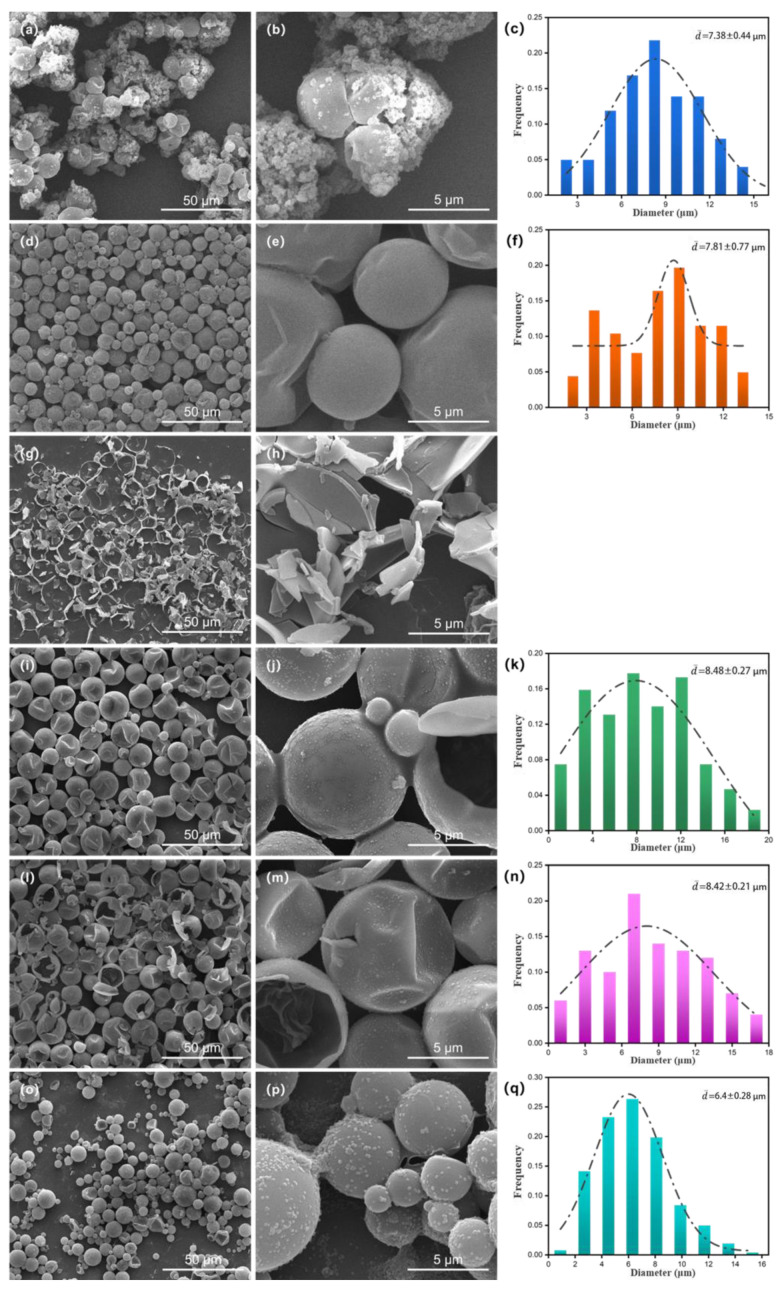
Microstructure and particle size distribution of microcapsules (**a**–**c**) pH3.5; (**d**–**f**) pH4.5; (**g**,**h**) pH5.5; (**i**–**k**) 1/1 (**l**–**n**) 4/1 (**o**–**q**) emulsifier dosage 15%.

Scanning electron microscopy (SEM) was used to observe the microcapsule morphology, as shown in Figure 2a,b,d,e,g,h. The microcapsule morphology is prepared when the pH is 3.5, 4.5 and 5.5. It can be seen from the figure that the microcapsule as a whole presents a regular spherical appearance. When the pH is 3.5, more irregular particles appear on the surface of microcapsules. As can be seen from the local magnification of Figure 2b, there is adhesion between the microcapsules. This is because when the initial pH is too low, the polycondensation rate of wall material prepolymers will accelerate, resulting in the self-polymerization of MUF resin. As a result, microcapsules adhere to each other and produce irregular particles on the surface. The wall material itself has no energy storage effect but occupies a part of the volume of the microcapsule. Therefore, the aggregation of excessively thick wall materials will reduce the overall latent heat value of the microcapsule and affect the energy storage effect. Figure 2d,e shows the microscopic morphology of the microcapsules prepared at pH 4.5. It can be seen from the figure that the surface of the microcapsules is smooth and well coated, and they are independent of each other without adhesion, indicating that the microcapsules have a good coating effect at pH 4.5. Figure 2g,h shows the microcapsule prepared at pH 5.5. At this pH value, a large number of microcapsules are broken, and it can be observed from the broken section of the wall material that the thickness of the wall material is very thin. This is because the polymerization speed of the prepolymer is very slow at this pH value, which leads to the instability in the synthesis process of the microcapsule. And the synthesis process has been accompanied by the shear force generated by the electric stirrer so that the microcapsule has a large number of broken phenomena and the core material cannot be effectively coated.

Figure 2d,e,i,j,l,m shows the microscopic morphology of microcapsules prepared with a core–wall ratio of 2/1, 1/1 and 4/1, respectively. When the core–wall material ratio is 1/1, there is a slight adhesion between microcapsules, and the cross-section of the wall material shows that the wall material of the microcapsules is thicker, as can clearly be seen in Figure 2j. This is because the number of prepolymers is more than the number of core molecules. When the core–wall ratio reaches 2/1, the surface impurities of microcapsules are significantly reduced, the surface impurities are smoother, there is no adhesion between each other and the dispersion is enhanced. As the core–wall ratio continues to increase to 4/1, the wall material cannot completely cover the core material, and a large number of microcapsules appear damaged, which will cause the core material to flow out from the damaged area, resulting in the leakage of the core material. Therefore, when the core–wall ratio is 2/1, the microcapsule has better coating and dispersion. SMA was selected as the emulsifier and emulsified at different dosages (5%, 10% and 15%). When the emulsifier dosage was 5%, n-octadecane could not be emulsified to form a stable emulsion and could not be successfully synthesized into microcapsules, so its microscopic morphology could not be observed.

Figure 2d,e,o,p shows the micromorphology of the microcapsules when the dosage is 10% and 15%. It can be seen that both of them show a relatively complete spherical appearance without fracture phenomenon, indicating that 10% emulsifier dosage is enough to emulsify the core material into a stable emulsion, so 10% is the optimal dosage. SMA, SDBS and SDS were, respectively, used for emulsification, and SMA could form a uniform and stable emulsion system for the core material, while the emulsion formed by SDBS and SDS would produce demulsification, which was not conducive to the synthesis of microcapsules in the next step. Therefore, the microcapsules prepared by using SMA emulsification to form emulsions had the best effect. Figure 2c,f,k,n,q shows the particle size diagram of microcapsules under different conditions. The microcapsules synthesized at pH 5.5 were broken too much, so it was impossible to use software to analyze their particle size. It can be seen that the average particle size of microcapsules was 6–8 μm, and different experimental conditions did not regularly affect the particle size of microcapsules. In addition, almost all microcapsules show different degrees of depression, which is because when the core material inside the microcapsule is cooled below the phase transition point, the core material will solidify and shrink in volume, while the area of the wall material basically remains unchanged, resulting in different degrees of microcapsule shrinkage [28].

### 2.2. Leak-Proof Analysis of Microcapsules

**Figure 3:**
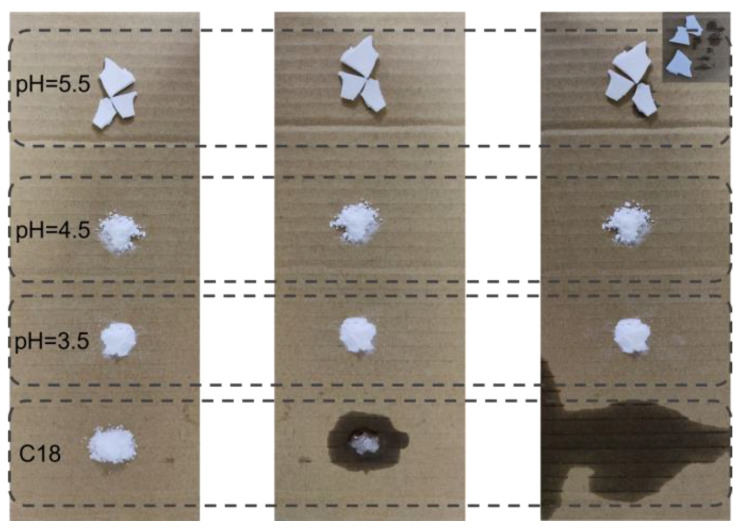
Leakage resistance of microcapsules.

The macro-leakage resistance of microcapsules at different pH values was tested. At 80 °C (higher than the melting temperature of n-octadecane), with the increase in time, the macro-leakage resistance of microcapsules is shown in Figure 3. From left to right is the leakage of the sample at 0 min, 10 min and 20 min. Among them, the microcapsules synthesized at pH 5.5 were blocky macroscopically. Combined with the SEM figure in the previous section, the microcapsules synthesized under this condition had poor coating properties and would solidify together when n-octadecane crystallized, resulting in the microcapsules being blocky macroscopically. As can be seen from Figure 3, n-octadecane began to melt gradually when heated at 80 °C for 10 min, resulting in leakage. At this time, the microcapsule materials could maintain good shape stability, and no leakage of n-octadecane was observed. When heated for 20 min, n-octadecane was completely melted. This instability will seriously affect the practical application of phase-change materials. While pH 3.5 and pH 4.5 microcapsules can still maintain good shape stability, no leakage of the internal core material was observed, but the bottom of pH 5.5 microcapsules also showed signs of leakage. Therefore, microcapsules with a pH of 3.5 and 4.5 have good leak-proof performance. Combined with the SEM diagram in the previous section, microcapsules have good microscopic morphology and excellent leak-proof performance when the pH is 4.5.

### 2.3. Chemical Structure of Microcapsules

**Figure 4:**
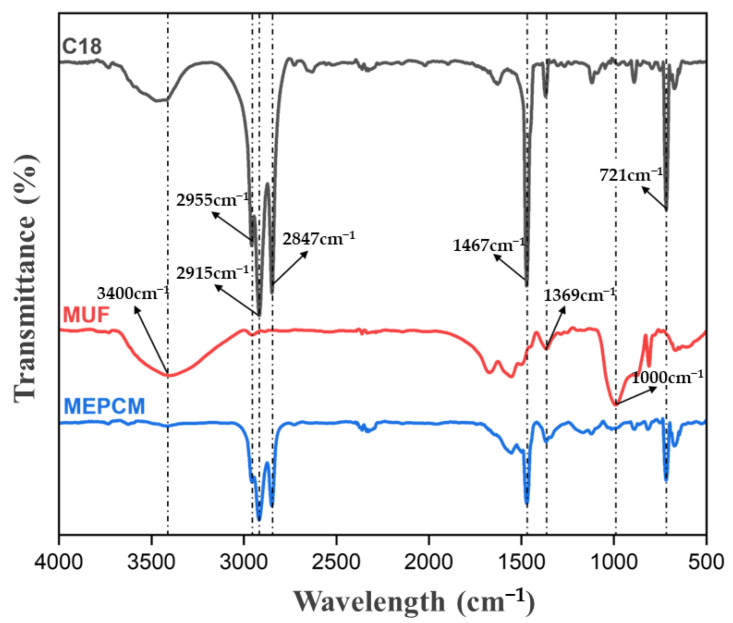
Infrared spectrum of n-octadecane, MUF wall material, microcapsule.

Fourier transform infrared spectroscopy was used to analyze the chemical structure of the microcapsules. The infrared spectra of MUF, n-octadecane and microcapsules are shown in Figure 4. Among them, the superposition of O-H and N-H tensile vibrations in MUF resin resulted in a strong and wide absorption peak near 3400 cm^−1^ [29]. The characteristic absorption peak at 1675 cm^−1^ is due to the C=O tensile vibration of urea in MUF resin. The tensile vibration of C-N is 1555 cm^−1^ [30]. The characteristic peak at 1365 cm^−1^ is caused by an O-H bending vibration. The characteristic peak at 1000 cm^−1^ is related to C-O tensile vibration. Due to the bending vibration of the triazine ring in MUF resin, there is a significant absorption peak at 1492 cm^−1^ and 812 cm^−1^. The above peaks are basically consistent with the IR spectra of the polymerization products of urea, melamine and formaldehyde. In the spectrum of n-octadecane, there are three characteristic peaks at 2955, 2915 and 2847 cm^−1^, which belong to the tensile vibrations of -CH, -CH_3_ and -CH_2,_ respectively. The peaks at 1467 cm^−1^ and 1369 cm^−1^ are caused by flexural vibrations of -CH_2_ and -CH_3_, respectively. In addition, the peak at 721 cm^−1^ is considered to be a typical peak of the alkyl (CH_2_) n (n ≥ 4) group [31]. In addition to the characteristic peaks of n-octadecane and MUF resin described above, no other characteristic peaks were observed in the spectrum of the microcapsule, indicating that the microcapsule has been successfully synthesized, the wall material is physically coated with the core material and there is no chemical reaction between the two.

**Figure 5:**
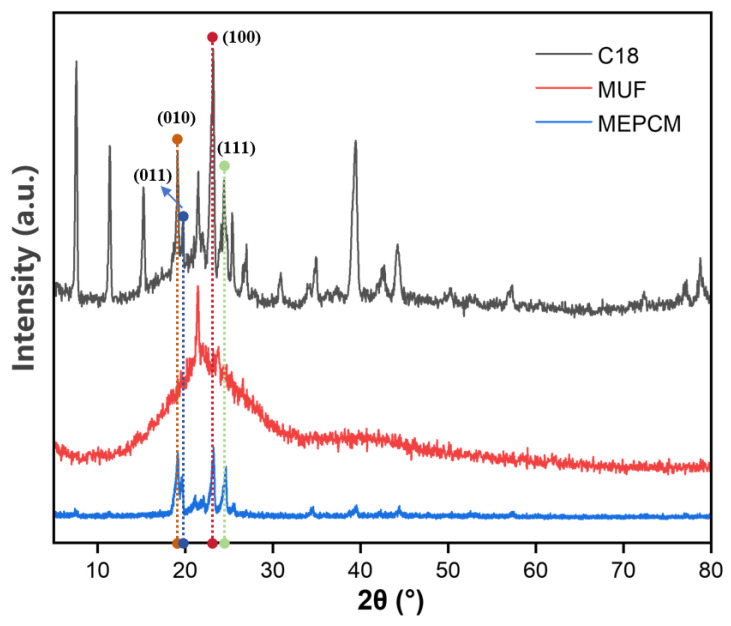
X-ray diffraction spectra of n-octadecane, MUF wall material and microcapsules.

Figure 5 shows the X-ray diffraction pattern of n-octadecane, MUF resin and microcapsules. The diffraction curve of n-octadecane has four distinct characteristic diffraction peaks at 19.3°, 19.7°, 23.4° and 24.7°, corresponding to the four characteristic crystal faces (010), (011), (100) and (111) in the triclinic system [32]. In the XRD pattern of MUF resin, a wide band can be observed only at 2θ = 15–30°, indicating that MUF resin is amorphous. In the XRD pattern of the microcapsule in Figure 5, only the diffraction peak of n-octadecane is observed, which is caused by the amorphous structure of the MUF shell. Combined with SEM and IR spectra, it can be said that n-octadecane is successfully coated in the microcapsule.

### 2.4. Thermal Properties Analysis of Microcapsules

**Figure 6:**
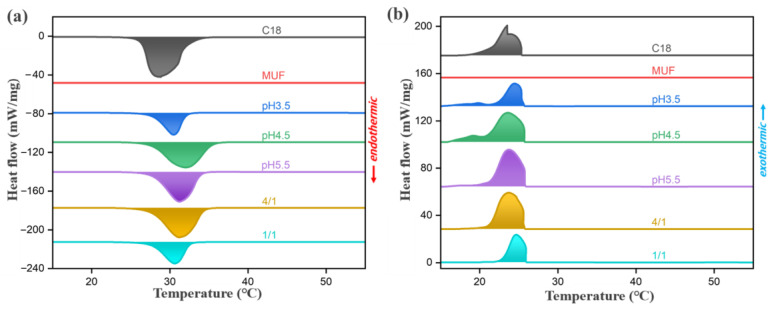
DSC curve of microcapsules: (**a**) melting, (**b**) cooling (−15–55 °C, heating/cooling rate = ±5 °C min^−1^).

The thermal properties of n-octadecane, MUF and microcapsules were measured using a differential scanning calorimeter, as shown in Figure 6, and the results are shown in Table 1. In the range of 5–55 °C, MUF does not have an absorption/exothermic peak, which indicates that the thermal effect of the microcapsule is mainly caused by the n-octadecane inside. As can be seen from Figure 6b, bimodal crystallization behavior occurred in both bulk n-octadecane and microcapsule samples during the crystallization process. The presence of two exothermal peaks is due to the fact that melt surface crystallization reduces molecular interactions before the complete crystallization of n-octadecane in bulk and microcapsules, resulting in the generation of metastable rotating phases [33,34,35]. The melting process has only a significant endothermic peak because both solid-metastable solids and metastable solid–liquids occur at close temperatures [36].

Table 1 shows that the latent heat of melting and crystallization (ΔH_m_ and ΔH_c_) of n-octadecane are 244.8 J g^−1^ and 244.2 J g^−1^, respectively. After encapsulation, the ΔH_m_ and ΔH_c_ of microcapsules decreased, because the wall material in microcapsules occupied a part of the volume but had no heat storage and release capacity, so the addition of wall material would reduce the volume of n-octadecane per unit volume of microcapsules, and thus reduce the latent heat. According to Formula (1), the encapsulation rate of microcapsules can be calculated. It can be found that with the decrease in pH, the encapsulation rate and latent heat value of microcapsules show a downward trend. This is because the polymerization of prepolymers is more rapid and sufficient under a low pH environment, and the self-polymerization of prepolymers may occur, which increases the volume ratio occupied by wall materials and thus reduces the latent heat value of microcapsules. Although the latent heat value of microcapsules is the highest at pH5.5, combined with the SEM image and anti-leakage performance test, the microcapsules at this time have a leakage risk and poor coating properties. When the pH was 4.5, the surface of the microcapsule was intact without leakage, the encapsulation rate reached 84.7%, and the latent heat value was still above 200 J g^−1^, maintaining a high level, and the overall performance was good. With the increase in the core–wall ratio, the encapsulation rate and latent heat value of the microcapsule gradually increase. However, when the core–wall ratio reaches 4/1, there are defects on the surface of the microcapsule, and the core material cannot be completely coated, resulting in the waste of a part of n-octadecane. Therefore, the core–wall ratio of 2/1 is a relatively ideal condition.

Our work was compared with the phase transition temperature and latent heat of microencapsulated phase change materials with C18 as the core material reported in recent years (Table 2). The result shows that our work is excellent.

This is example 1 of an equation:

(1) $$E_{\mathrm{en}}\;=\frac{\Delta H_{m,MEPM}}{\Delta H_{m,\;\;PCM}}\;\times \;100\%$$

where Δ*H_m,PCM_*, Δ*H_m__,MEPCM_* are the latent heat of the melting of massive n-octadecane and n-octadecane microcapsules, respectively.

### 2.5. Thermal Stability Analysis of Microcapsules

**Figure 7:**
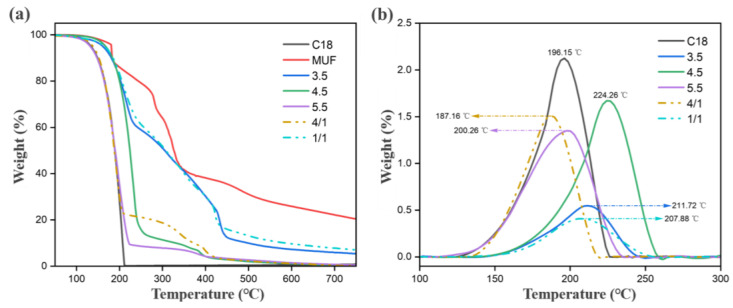
Thermogravimetric curve of microcapsules (**a**) TGA (**b**) DTG.

The range of applications and uses of microcapsules largely depend on their thermal stability [37,38]. The thermal degradation behavior of n-octadecane, MUF resin and microcapsules was studied using TGA, and the TGA and DTG curves are shown in Figure 7a,b. The TGA curve shows that n-octadecane exhibits a typical one-step degradation curve under heating conditions, that is, from 99 °C to 221 °C, the weight decomposition of n-octadecane approaches 100%, which means that n-octadecane is almost completely decomposed. In contrast, the TGA curve of microcapsule samples showed a two-step thermal degradation process. The evaporation of n-octadecane and the degradation of MUF resin could be clearly seen in the TGA curve of the microcapsule. The evaporation of n-octadecane led to the first step of degradation, which occurred between 131 and 66 °C. MUF is broken, so the weightlessness of microcapsules in this step is significant. After this, a further weight loss occurs between 270 and 335 °C, which is due to the decomposition of the MUF shell. As can be seen from the DTG curve, due to the protection of the MUF shell on n-octadecane, the decomposition of n-octadecane in microcapsules has a temperature lag compared with that of n-octadecane in bulk. Therefore, the thermal stability of microcapsules is improved compared with that of bulk n-octadecane. When the pH is 4.5, the maximum degradation rate temperature reaches 224.3 °C, which is 28.1 °C higher than bulk n-octadecane.

With the increase in the core–wall ratio and pH during the synthesis of microcapsules, the wall material cannot provide better barrier protection for the inner core material, and the thermal stability of microcapsules also decreases. In addition, the TGA curve of the microcapsule synthesized under the conditions of pH 3.5 and core–wall ratio of 1/1 shows that there is still about 5% weight residue when the temperature reaches 750 °C, which is because the MUF resin as the wall material will be left after high-temperature heating. The wall material synthesized under this condition accounts for a relatively large proportion of the microcapsule wall material, and the amount of residual carbon after high-temperature decomposition is correspondingly more. The operating temperature of the microcapsule is below 100 °C, at which time the microcapsule loses only 0.12% of its volume weight. From another perspective, the initial decomposition temperature of the microcapsule is significantly higher than its operating temperature. Therefore, it can be determined that the microcapsule has a high thermal stability in its operating temperature range.

**Table 2:** Comparison with the literature on phase-change microcapsules with n-octadecane as core material.

Core Component	Melting		Cooling		Ref
	T_pm_/(°C)	ΔH_m_/(J/g)	T_pc_/(°C)	ΔH_c_/(J/g)	
C18	20.5	184.6	30.7	185.6	[39]
C18/C28	51.9	187.9	48.8	187.7	[40]
C18	29.0	114.5	23.1	118.8	[41]
C18/C22	39.8	100.8	-	-	[42]
C18	29.9	116.5	27.0	124.7	[43]
C18/C-PODMA	24.1	111.6	16.7	112.6	[44]
C24-C18	26.0	156.4	26.0	152.8	[45]
C19-C18	26.4	114.1	26.5	113.4	[46]
C18	30.6	171.9	25.3	172.4	[47]
C18	-	125.9	-	-	[48]
C18	32.0	207.3	23.7	181.6	This work

## 3. Materials and Methods

### 3.1. Materials

N-octadecane (C18) (AR), melamine (AR), formaldehyde solution (37–40 wt%) and urea were purchased (AR) from Shanghai Aladdin Biochemical Technology (Shanghai, China) Co., Ltd. Styrene–Maleic anhydride (SMA) (95%) was purchased from Nanjing Yinxin (Nanjing, China) Chemical Co., Ltd. Sodium hydroxide (AR), triethanolamine (AR) and urea (AR) were also purchased from Aladdin Biochemical (Shanghai, China) Technology Co., Ltd.

### 3.2. Preparation of n-Octadecane-SMA Core Material

Add 0.05 g NaOH into the three-neck flask, stir with 40 mL deionized water until the NaOH is completely dissolved, then add 0.25 g styrene–maleic anhydride copolymer (SMA) into the flask and place the three-neck flask in a constant temperature water bath at 90 °C. Use an electric mixer at 600 rpm to stir until the SMA is completely dissolved, and then cool to room temperature to obtain the SMA solution. The pH of the SMA solution was adjusted to 3.5~5.5 with 10 wt% citric acid solution, and then 5 g n-octadecane was added to the SMA solution and transferred to a constant temperature water bath at 70 °C. When the n-octadecane was completely melted, a homogenized n-octadecane emulsion was obtained by emulsifying at 13,000 rpm for 30 min using a high-speed homogenizer.

### 3.3. Preparation of MUF Shell

Add 3.12 g formaldehyde solution to the three-mouth flask, add 19.9 g deionized water, adjust the pH to 8.5 with 10 wt% triethanolamine solution and then add 0.69 g melamine. The three-neck flask was transferred to a constant temperature water bath at 75 °C and the solution was stirred with an electric mixer at 600 rpm until the solution was clarified. Then, 0.58 g urea was added and the reaction was continued for 20 min under the above experimental conditions to obtain the MUF prepolymer solution.

### 3.4. Synthesis of Microcapsules

The n-octadecane emulsion was placed in a constant temperature water bath at 70 °C, and the MUF prepolymer solution was slowly added to the n-octadecane emulsion with an electric mixer at the stirring speed of 600 rpm. After the drip was completed for 15 min, the reaction was carried out at 70 °C and 600 rpm for 3 h, and then the reaction system was taken out and left at room temperature for 12 h. Due to the low density of n-octadecane, the upper layer is a n-octadecane microcapsule. The impurities in the microcapsule are removed by rinsing with deionized water and ethanol at 80 °C 3–5 times, and then the microcapsule is dried in a vacuum drying oven at 80 °C for 24 h to obtain n-octadecane microcapsule powder.

### 3.5. Characterization

#### 3.5.1. Scanning Electron Microscopy (FE-SEM)

A scanning electron microscope (SEM, FEI Quanta 200, Fremont, CA, USA) was used to observe the micromorphology of microcapsules. Image J (v 1.8.0) software was used to analyze the size distribution and average particle size of microcapsules.

#### 3.5.2. Fourier Transform Infrared Spectroscopy (FTIR)

The functional groups of the sample were determined using Fourier transform infrared spectroscopy (FTIR, IRAffinity-1S, SHIMADZU, Tokyo, Japan) with a wavelength range of 500–4000 cm^−1^. The MUF, paraffin and paraffin/MUF microcapsules obtained under the best conditions were pressed into ultra-thin particles.

#### 3.5.3. Thermogravimetric Analysis (TGA)

Thermogravimetric analysis of MUF, paraffin and paraffin/MUF microcapsules obtained under optimal conditions was performed using a thermogravimetric analyzer (TGA, NETZSCH, TG209 F3, Selb, Germany). Each sample (~8 mg) was measured with dry pure N_2_ (50 mL min^−1^) at elevated temperatures (25~750 °C, 10 °C min^−1^).

#### 3.5.4. X-ray Diffractometer (XRD)

X-ray diffractometry (XRD, ARL EQUINOX 1000, Waltham, MA, USA) was used to characterize the crystal structure of the sample with a scanning angle of 5–80 ° and a scanning speed of 4 °/min.

#### 3.5.5. Differential Scanning Calorimetry (DSC)

A differential scanning calorimeter (DSC4000, PerkinElmer, Waltham, MA, USA) (−15–55 °C, heating/cooling rate = ±5 °C min^−1^) was used to analyze the phase transition characteristics of paraffin and paraffin/MUF microcapsules. Nitrogen was used as a protection and purification gas. For DSC analysis, each sample (~6 mg) was measured in an aluminum crucible. The melting temperature (T_m_) and latent heat of melting (ΔH_m_) were determined by the heating scan, and the crystallization temperature (T_c_) and latent heat of crystallization (ΔH_c_) were determined by the cooling scan.

## 4. Conclusions

Using the pH of the core material and the core-to-wall ratio as entry points for studying the performance of phase-change microcapsules, octadecane core layer phase-change microcapsules with a MUF shell were prepared by the in situ polymerization method. The emulsifier content and the initial pH value of the core material have a decisive role in the shape and encapsulation efficiency of the microcapsule. The content of the core material and the core–wall ratio of the phase-change microcapsule also have a huge impact on the latent heat and structural stability of the microcapsule. By adjusting these parameters in the experimental system, we were surprised to find that when the pH = 4.5 and the core–wall ratio is 2/1, the prepared MUF shell-coated octadecane phase-change microcapsule has the most ideal performance. Specifically, the phase-change microcapsule at this time has a perfect spherical core–shell structure, the encapsulation efficiency is 84.7% higher than that of the unpacked octadecane phase-change material and it also has excellent thermal stability, shape stability and thermal conductivity (0.1288 W/m·K). We also found that with the decrease in the pH and core-to-wall ratio, although the leakproof and thermal stability of microcapsules were improved, their heat storage capacity decreased. When the pH is 4.5 and the core-to-wall ratio is 2/1, the latent heat of the prepared MUF shell-coated octadecane phase-change microcapsules can still reach 207.3 J g^−1^.

## Figures and Tables

**Table 1:** Thermal properties of microcapsules.

Sample	Melting				Cooling				E_en_ (%)
	T_om_/(°C)	T_pm_/(°C)	T_em_/(°C)	ΔH_m_/(J/g)	T_oc_/(°C)	T_pc_/(°C)	T_ec_/(°C)	ΔH_c_/(J/g)	
C18	26.4	28.8	35.7	244.8	25.4	23.5	17.1	244.2	
MUF	-	-	-	-	-	-	-	-	-
pH3.5	23.9	30.5	33.4	120.9	25.7	24.5	15.9	88.2	49.4
pH4.5	24.4	32.0	37.0	207.3	25.8	23.7	15.9	181.6	84.7
pH5.5	24.8	31.3	35.1	219.8	25.8	23.7	16.7	208.8	89.8
4/1	23.7	31.2	35.6	192.4	25.9	23.7	18.9	177.4	78.6
1/1	24.5	30.6	33.5	140.2	26.0	24.7	21.4	138.1	52.3

## Data Availability

Data will be made available upon request.
